# Self-expandable valve-in-valve transcatheter aortic valve implantation for a failed sutureless Perceval bioprosthesis: a case report

**DOI:** 10.1093/ehjcr/ytag095

**Published:** 2026-02-04

**Authors:** Strahil K Vasilev, Ivo S Petrov, Zoran I Stankov, Dayana K Emanuilova

**Affiliations:** Department of Cardiology, Angiology and Electrophysiology, ‘Acibadem City Clinic’ University Hospital - Vitosha, 1 Konstantin Pomyanov Street, Sofia 1700, Bulgaria; Department of Cardiology, Angiology and Electrophysiology, ‘Acibadem City Clinic’ University Hospital - Vitosha, 1 Konstantin Pomyanov Street, Sofia 1700, Bulgaria; Department of Cardiology, Angiology and Electrophysiology, ‘Acibadem City Clinic’ University Hospital - Vitosha, 1 Konstantin Pomyanov Street, Sofia 1700, Bulgaria; Department of Diagnostic Imaging, ‘Acibadem City Clinic’ University Hospital - Vitosha, Sofia 1700, Bulgaria

**Keywords:** Valve-in-valve TAVI, Case report, Sutureless aortic valve, Perceval bioprosthesis, Self-expandable transcatheter heart valve, Multidetector computed tomography, Structural valve degeneration

## Abstract

**Background:**

Early structural degeneration of sutureless Perceval bioprostheses is uncommon but may result in severe haemodynamic compromise and heart failure. Valve-in-valve transcatheter aortic valve implantation (ViV-TAVI) represents a less invasive therapeutic option in high-risk patients.

**Case summary:**

A 78-year-old woman presented with progressive dyspnoea and lower-extremity oedema 3 years after surgical aortic valve replacement with a Perceval bioprosthesis. Transthoracic echocardiography revealed severe mixed prosthetic valve dysfunction with markedly elevated transvalvular gradients. Owing to prohibitive surgical risk, comprehensive preprocedural planning with echocardiography and multidetector computed tomography was performed to assess annular dimensions and coronary anatomy. A self-expandable ViV-TAVI was successfully performed, guided by fluoroscopic visualization of the Perceval inflow ring.

**Discussion:**

This case highlights the importance of understanding sutureless valve geometry and meticulous computed tomography–based planning when performing ViV-TAVI with a self-expandable transcatheter valve.

Learning points

- Self-expandable valve-in-valve transcatheter aortic valve implantation is a feasible treatment option for failed sutureless Perceval prostheses in selected high-risk patients.
- Understanding Perceval valve geometry and fluoroscopic identification of the inflow ring is essential for accurate transcatheter valve positioning.
- Preprocedural computed tomography-based assessment of coronary anatomy and valve-to-coronary distance is critical to reduce the risk of coronary obstruction.

## Introduction

Sutureless aortic valve prostheses such as the Perceval bioprosthesis were developed to shorten operative times and facilitate minimally invasive surgical aortic valve replacement while preserving favourable haemodynamic performance. Despite good mid-term outcomes, early structural valve degeneration has been increasingly reported and represents a challenging clinical scenario, particularly in elderly patients with high surgical risk. Valve-in-valve transcatheter aortic valve implantation (ViV-TAVI) has emerged as an alternative treatment option for degenerated surgical bioprostheses, reducing perioperative morbidity and mortality compared with redo surgery. However, ViV-TAVI in sutureless valves requires specific anatomical considerations related to prosthesis design, anchoring zones, and coronary obstruction risk. This case illustrates the feasibility and technical aspects of performing self-expandable ViV-TAVI in a failed Perceval prosthesis using detailed multimodality imaging guidance.^1–3^

## Summary figure

**Figure:**
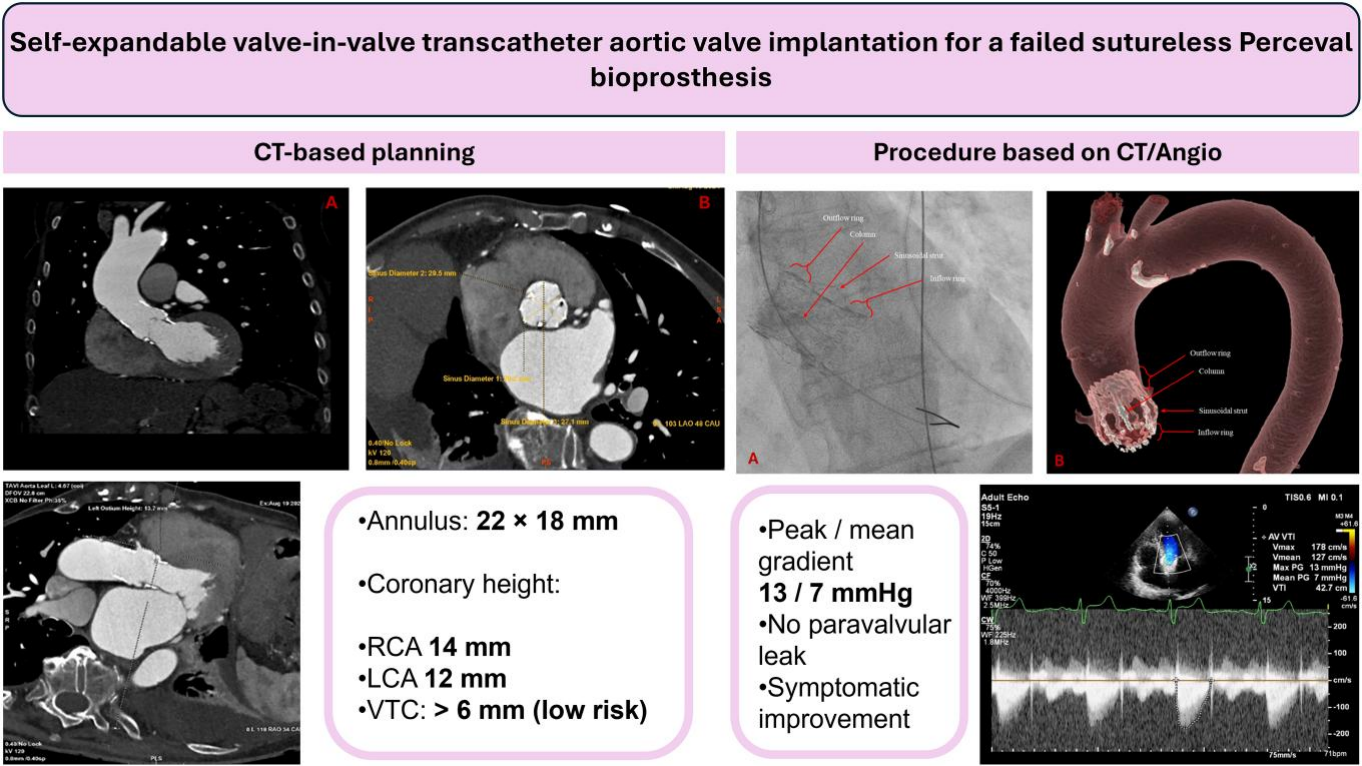


## Case presentation

A 78-year-old woman was admitted with progressive dyspnoea on minimal exertion and worsening lower-extremity oedema. On presentation, she was orthopneic but haemodynamically stable, with a heart rate of 70 beats per minute and a blood pressure of 129/48 mmHg, without inter-arm difference. Cardiac auscultation revealed a Grade 3/6 systolic ejection murmur at the second right intercostal space radiating to the carotid arteries. Physical examination demonstrated pallor, reduced breath sounds at the right lung base, and marked bilateral peripheral oedema.

Three years earlier, the patient had undergone surgical aortic valve replacement with implantation of a sutureless Perceval bioprosthesis and concomitant decalcification of the anterior mitral leaflet. Her medical history included long-standing hypertension, Type 2 diabetes mellitus, chronic anaemia, and multiple drug allergies.

Transthoracic echocardiography demonstrated severe degeneration of the aortic bioprosthesis with mixed stenotic and regurgitant dysfunction. Peak and mean transvalvular gradients were 88 and 53 mmHg, respectively, with a calculated aortic valve area of 0.50 cm². Additional findings included moderate-to-severe mitral regurgitation, moderate tricuspid regurgitation, preserved left ventricular ejection fraction, reduced right ventricular systolic function, severe pulmonary hypertension (estimated systolic pulmonary artery pressure of 70 mmHg), and a right-sided pleural effusion.

**Figure 1:**
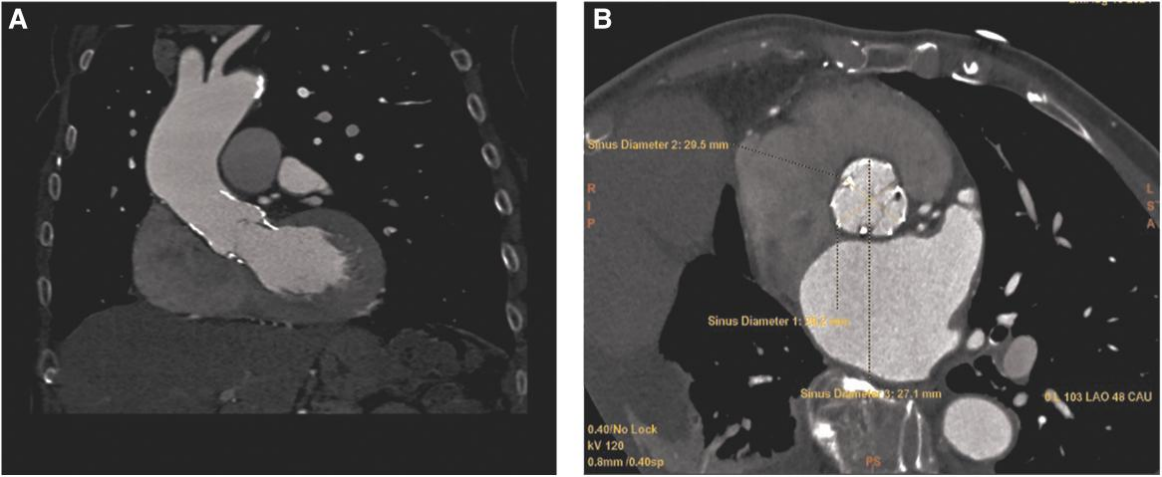
Preprocedural multidetector computed tomography planning. (*A*) Measurement of the intended landing zone corresponding to the inflow ring of the sutureless Perceval bioprosthesis. (*B*) Assessment of aortic root dimensions, including annular geometry, to guide valve sizing for valve-in-valve transcatheter aortic valve implantation.

**Figure 2:**
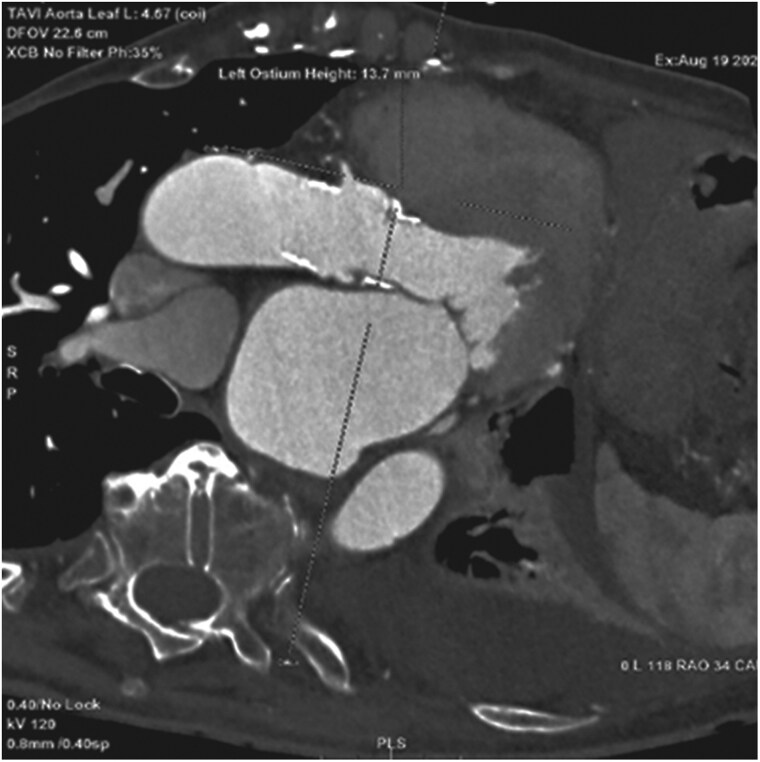
Multidetector computed tomography assessment of coronary anatomy. Oblique reconstruction demonstrating the left coronary artery take-off and measurement of coronary ostial height relative to the aortic annulus, allowing evaluation of valve-to-coronary distance prior to valve-in-valve implantation.

Given the prohibitive surgical risk (EuroSCORE II 25.2%, STS-predicted mortality and morbidity 30.4%), the Heart Team recommended ViV-TAVI. Multidetector computed tomography (CT) was performed for procedural planning, revealing annular dimensions of 22 × 18 mm, consistent with a previously implanted Perceval size M (21–23 mm). The right and left coronary artery heights were 14 and 12 mm above the annulus, respectively, with an adequate valve-to-coronary distance (*Figures 1* and *2*).

**Figure 3:**
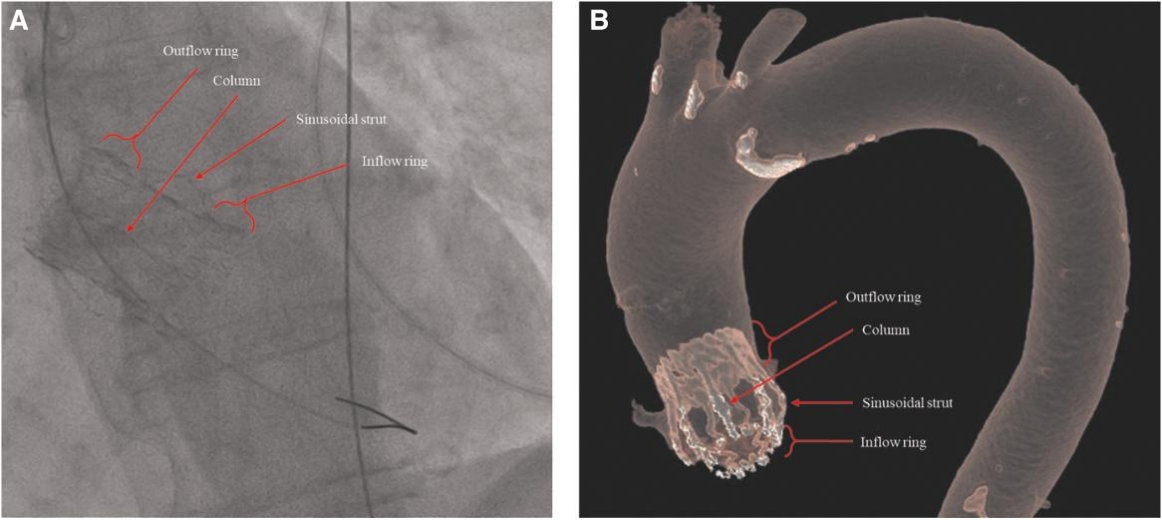
Anatomical characteristics of the sutureless Perceval bioprosthesis. (*A*) Fluoroscopic view showing the radiopaque inflow ring of the Perceval valve, used as a landmark for transcatheter valve positioning. (*B*) Corresponding computed tomography reconstruction illustrating the inflow and outflow rings and supporting stent frame of the Perceval prosthesis.

A minimalist transfemoral TAVI approach was employed. A temporary pacing lead was placed via the right antecubital vein, with primary arterial access obtained through the right femoral artery and a 5-Fr right radial access used for aortic root angiography. The Perceval prosthesis was crossed within the outflow ring, followed by balloon predilatation using a 20 × 40 mm balloon under rapid ventricular pacing. A 26 mm self-expandable Evolut R valve (Medtronic, Minneapolis, MN, USA) was then successfully implanted, guided by fluoroscopic visualization of the radiopaque Perceval inflow ring to ensure accurate positioning (*Figure 3*; see Supplementary material online, *Videos S1*–*S4*).

## Discussion

Sutureless aortic valve prostheses, such as the Perceval valve, were developed to facilitate surgical aortic valve replacement by reducing aortic cross-clamp and cardiopulmonary bypass times while maintaining favourable haemodynamic performance.^3^ Despite these advantages, early structural valve degeneration has been increasingly reported, posing a therapeutic challenge, particularly in elderly and high-risk patients.^4–7^

Valve-in-valve transcatheter aortic valve implantation has emerged as a less invasive alternative for failed surgical bioprostheses.^2–4^ However, ViV-TAVI in sutureless valves requires careful consideration of prosthesis geometry and anchoring mechanisms. Unlike conventional stented bioprostheses, the Perceval valve consists of a self-expanding nitinol frame with distinct inflow and outflow rings connected by vertical struts.^4^ The fluoroscopically visible inflow ring represents the primary anchoring and landing zone for transcatheter valve deployment.

Meticulous preprocedural planning with multidetector CT is essential in this setting. Accurate assessment of annular dimensions, coronary artery heights, and valve-to-coronary distance is critical to minimize the risk of coronary obstruction, one of the most feared complications of ViV-TAVI.^4–8^ A valve-to-coronary distance greater than 6 mm is generally considered low risk and was confirmed in the present case, allowing safe progression to intervention.

Most reported cases of ViV-TAVI in failed Perceval valves have utilized balloon-expandable transcatheter heart valves.^7–10^ Although balloon-expandable devices provide precise deployment, concerns remain regarding incomplete expansion, elevated residual gradients, and paravalvular leak when implanted within the elastic nitinol frame of a sutureless prosthesis.^4^ In this case, a self-expandable valve was selected to provide continuous radial force, improved conformability to the Perceval frame, and favourable haemodynamic performance with low residual gradients.

Fluoroscopic identification of the Perceval inflow ring proved essential for accurate valve positioning and served as a reliable landmark during deployment. This technical aspect underscores the importance of understanding sutureless valve architecture when performing ViV-TAVI and supports the feasibility of self-expandable valve implantation in this specific anatomical context.^4^

## Outcome and follow-up

**Figure 4:**
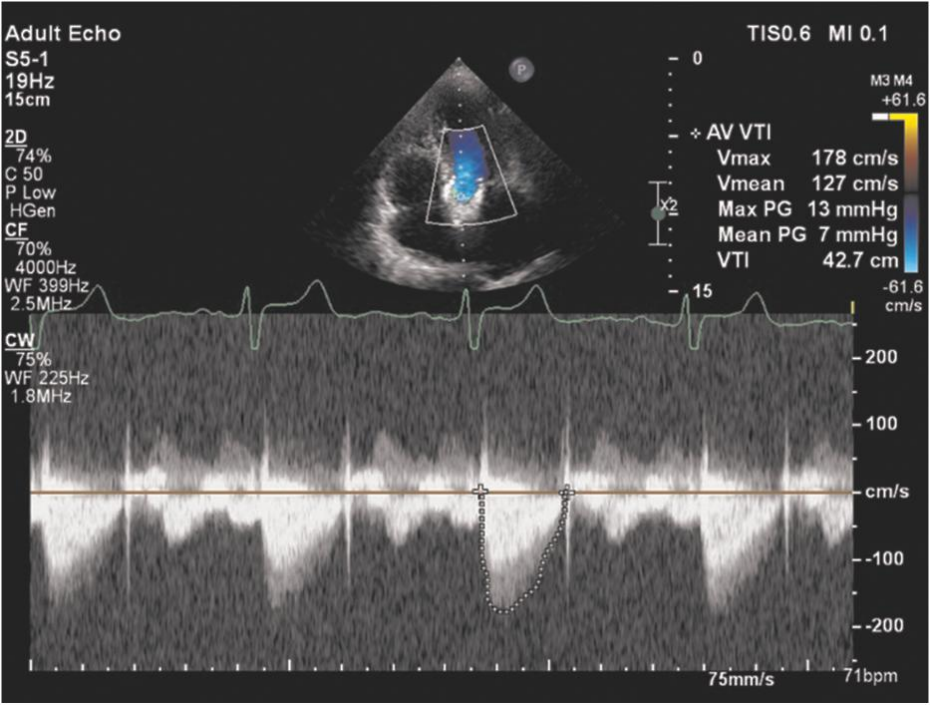
Follow-up transthoracic echocardiography after valve-in-valve transcatheter aortic valve implantation. Continuous-wave Doppler across the aortic valve demonstrates low peak and mean transvalvular gradients with no evidence of paravalvular regurgitation.

The postprocedural course was uncomplicated, and the patient was discharged on the second day after the intervention. At 6-month follow-up, she demonstrated marked clinical improvement with a significant reduction in heart failure symptoms. Transthoracic echocardiography revealed a well-functioning transcatheter aortic valve with low peak and mean gradients (13/7 mmHg), absence of paravalvular leak, and improvement of concomitant mitral regurgitation to mild-to-moderate severity (*Figure 4*).

## Conclusions

Valve-in-valve transcatheter aortic valve implantation represents a feasible treatment option for failed sutureless Perceval bioprostheses in selected high-risk patients. Careful preprocedural planning with multidetector CT, accurate assessment of coronary anatomy, and a thorough understanding of Perceval valve geometry are essential for procedural success. Fluoroscopic identification of the inflow ring facilitates precise positioning of a self-expandable transcatheter valve and favourable haemodynamic outcomes.

## Patient perspective

Before the procedure, I experienced severe shortness of breath and was unable to perform my usual daily activities. I was worried about undergoing another heart operation and the associated risks. After the transcatheter valve procedure, my breathing improved significantly, and I was able to return to my normal routine. I am grateful for the minimally invasive treatment option and the care provided by the medical team.

## Lead author biography


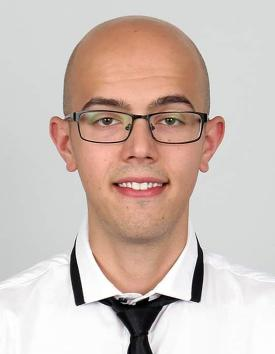


Strahil K. Vasilev, MD, is a cardiology resident at the Department of Cardiology, Angiology and Electrophysiology, Acibadem City Clinic University Hospital, Sofia, Bulgaria. He obtained his medical degree from the Medical University of Sofia. His clinical and research interests focus on structural heart disease, transcatheter valve interventions, coronary interventions, and advanced cardiovascular imaging. Dr Vasilev is actively involved in the management of high-risk patients undergoing transcatheter aortic valve implantation and valve-in-valve procedures. He has a strong interest in clinical research and case-based education in interventional cardiology.

## Supplementary Material

ytag095_Supplementary_Data

## Data Availability

The data underlying this article are available within the article and its Supplementary material. Additional anonymized data will be shared on reasonable request to the corresponding author.

## Supplementary material

**Consent:** The authors confirm that written informed consent for submission and publication of this case report, including images and associated text, has been obtained from the patient in accordance with the COPE guidelines.
